# An edge‐based interface‐tracking method for multiphase flows

**DOI:** 10.1002/fld.5144

**Published:** 2022-10-19

**Authors:** Leonardo Chirco, Stéphane Zaleski

**Affiliations:** ^1^ Sorbonne Université and CNRS Institut Jean Le Rond d'Alembert Paris France; ^2^ Institut Universitaire de France Paris France

**Keywords:** front‐tracking, interface tracking, level‐set, two‐phase flows

## Abstract

We propose a novel class of edge‐based interface‐tracking (EBIT) methods in the field of multiphase flows for advecting the interface. The position of the interface is tracked by marker points located on the edges of the underlying grid, making the method flexible with respect to the choice of spatial discretization and suitable for parallel computation. In this article we present a simple EBIT method based on two‐dimensional Cartesian grids and on a linear interface representation.

## Graphical abstract

We propose a class of methods to track the interface with a set of markers attached to the grid lines. The method works on any kind of grid, can be easily parallelized, and the addition or removal of markers is straightforward. We focus on the special 2D case where the intersections are at most two per square face of the grid and the interpolation between the marker points is linear.

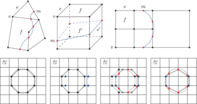

## 1 INTRODUCTION

Many methods for following an interface or front exist, the simplest and most popular being the front‐tracking, the level‐set and the volume‐of‐fluid method.^1^ In this article we first consider a new class of methods, which could be called edge‐based interface‐tracking (EBIT) methods. In these methods, the basic information about the front position is known or “tracked” by the position of marker points, which makes the method a kind of front‐tracking. However, the additional requirement is that the markers are located on the edges of the underlying grid. When the connecting interface lines between the marker points are linear, the method bears an obvious similarity with the volume‐of‐fluid method of Piecewise Linear Interface Calculation type (PLIC‐VOF). Finally, since the position of the markers gives an explicit information about the distance of the vertices of the underlying grid to the interface, it is a kind of distance information as in the level‐set method, where the implicit definition of the interface is given by a function as close as possible to the signed distance function. In particular, a linear interface has the same representation using EBIT and level‐set methods.

**FIGURE 1:**
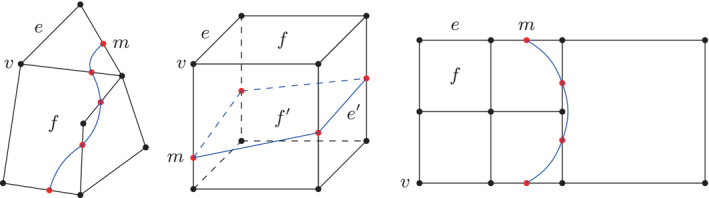
Schematics of edge‐based interface‐tracking. (Left) On an unstructured planar grid formed of edges e (black lines), vertices v (black dots), and faces f (polygons delimited by black lines), the interface passes through the markers m (red dots). (Center) On a regular cuboid volume grid, with again edges e (black lines), vertices v (black dots), and faces f (squares delimited by black lines), the interface passes through the markers m (red dots). The markers m form the vertices of the surface grid tracking the interface, with edges $e^{\prime }$ and faces $f^{\prime }$ on the latter grid. The faces are in general non‐planar. (Right) The leaf cells on a quadtree grid form a particular type of unstructured grid. The edges can again be the location of marker points. [Colour figure can be viewed at wileyonlinelibrary.com]

Several prior works have attempted a combination of pairs of the three main methods and may result in methods similar to this one, such as the combination of markers and VOF^2^ or the combination of level‐set and front‐tracking.^3^ However, the EBIT method adds the simplifying requirement that only the position of the markers on the grid lines or grid edges needs to be known. This is true both in 2D and 3D and whatever the grid type, structured, unstructured, or hierarchical/quadtree, see Figure 1. The use of iso‐faces to perform the advection of interfaces on general meshes consisting of arbitrary polyhedral cell is the core of the *isoAdvector* algorithm as well, see Reference 4. Perhaps the most important advantage of EBIT methods is that they allow for almost automatic parallelization. In fact, since the marker points are constrained to move along the grid edges, their re‐distribution among processes follows naturally that of the grid cells. Another potential advantage is that as the grid is adapted, refined, or unrefined the front is adapted consistently. Finally, since information about the connectivity of the marker points does not need to be stored (it can be reconstructed and is thus known implicitly) the addition or removal of points or grid cells is easier than in traditional front‐tracking.^5^ In this article we focus on a special case of EBIT methods, the Semushin method, in which the underlying grid is a 2D square grid, the intersections are at most two per square edge of the grid and the interpolation between the marker points is linear. This is clearly a “bare bones” version of the EBIT method and is inspired by Semushin's preprint^6^ and by personal communications received from its author. This article is organized as follows. The method is described is Section 2 and then in Section 3.2 the numerical results are presented. Finally, the conclusions are given in the last section.

## 2 THE SEMUSHIN METHOD

In Semushin's method for tracking the interface, the reference phase is enclosed by a set of marker points placed on the grid lines. The advection of the interface is done by moving these points along the grid lines. Thanks to this constraint, the n‐dimensional advection algorithm can be split into a succession of n times the one‐dimensional scheme, one for each direction.

The equation of motion for the interface point is

(1) $$\frac{dx}{dt}=u,$$

that can be integrated as

(2) $$x=x_{0}+\int_{t_{0}}^{t}u(x(t^{\prime }),t^{\prime })dt^{\prime },$$

where the initial position $x_{0}$ is known. For the sake of simplicity, in this work we use a first‐order explicit Euler method such that $x=x_{0}+u_{0}\Delta t=x_{0}+\Delta x$.

**FIGURE 2:**
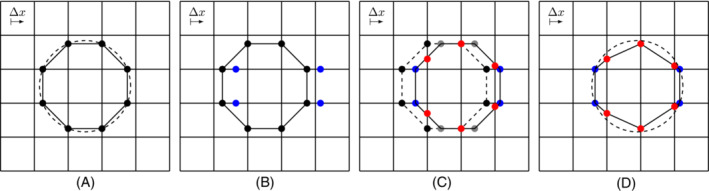
The steps of the one‐dimensional advection scheme of Semushin's method. (A) Initial markers position for the dashed circle; (B) advection of the (blue) points aligned with the velocity; (C) fictitious advection of the unaligned (gray) points and (red) intersections; (D) final position of the markers and interface [Colour figure can be viewed at wileyonlinelibrary.com]

Now, we describe the simple one‐dimensional advection algorithm used, see Figure 2. We recall that we study a two‐dimensional problem, admit at most two interface intersections (and then markers) per face (edge in 2D) of the grid, and that the interpolation between the marker points is linear. The extension to three‐dimensional problems or unstructured grids is straightforward, see Figure 1. The points placed on the grid lines aligned with the velocity are called *aligned* points, while the remaining ones are *unaligned*. Starting from the initial configuration (Figure 2A), the new position of the aligned points (Figure 2B) is directly obtained by integrating (2). To place the unaligned points (in this example on the vertical grid lines), we first advect them using the same Equation (2) obtaining the *fictitious* gray points in Figure 2C. Finally, the new position of the unaligned points (in red in Figure 2C) is obtained by connecting with a segment either one blue and one gray point or two consecutive gray points and by finding the intersection with the grid lines. The position of the points and of the interface after the advection along the x‐direction is shown in Figure 2D.

## 3 RESULTS

We define the surface error $E_{area}(t)$ between the total area of the reference phase at the initial time $t_{0}$ and time t as

(3) $$E_{area}=\frac{\left|A(t)-A(t_{0})\right|}{A(t_{0})}.$$

We define the shape error, in a $L^{\infty }$ norm, as the maximum distance between any marker point $x_{i}$ on the interface and the corresponding closest point on the analytical shape as

(4) $$E_{shape}=\underset{i}{\max}|\text{dist}(x_{i})|.$$

We recall that for a circle centered in $\left(x_{c},y_{c}\right)$ and radius R, we have $\text{dist}(x_{i})=\sqrt{{\left(x_{i}-x_{c}\right)}^{2}+{\left(y_{i}-y_{c}\right)}^{2}}-R$. The order of convergence of the method is computed by comparing the errors on successively refined grids as

(5) $$\text{order}=\log_{2}(E(h)/E(h/2)),$$

where E(h) is the norm of the error on the grid with spacing h, with respect to the exact solution. We perform three well‐known tests to evaluate the accuracy of interface advecting methods.^7^

### 3.1 Translation with uniform velocity

In the first test a circular shape of radius r=0.15 and center (0.25,0.75) is placed inside the unit box. The box is meshed with $N_{x}\times N_{x}$ square cells of size $h=1/N_{x}$, where $N_{x}=64,128,256,512$. A uniform and constant velocity field (u,v) with u=−v is imposed in the box, so that the reference phase is advected along the diagonal of the box. After one time unit, the velocity field is reversed and the circular fluid body should return to its initial position with no distortion, allowing error measurement with (3) and (4). For this test, we employ two constant *CFL* numbers CFL=uΔt/h, where Δt is the time step. For example, if CFL=1, the circle is displaced of exactly one grid spacing per time step, while if CFL<1, the circle advances only by a fraction of the grid spacing.

**FIGURE 3:**
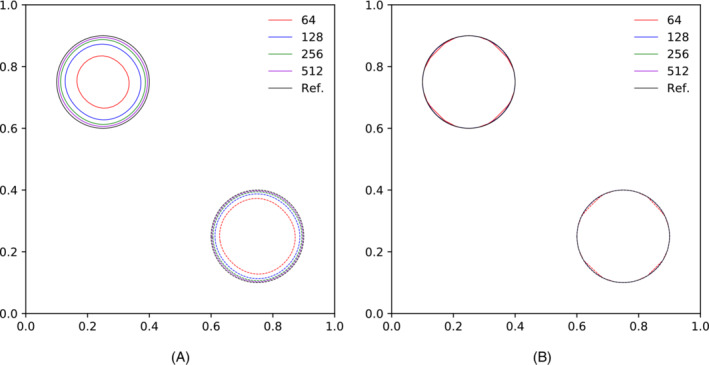
Final circular shape (solid line) and after half diagonal translation (dashed line). (A) CFL=0.125; (B) CFL=1 [Colour figure can be viewed at wileyonlinelibrary.com]

In Figure 3, the position of the reference phase is shown after two full diagonal translations (solid line) and one (dashed line). When using the coarser grids, the circular shape is shrunk radially. In Table 1 we report the surface error $E_{area}$, the shape error $E_{shape}$, and order of convergence for two complete translations along the main diagonal, at different resolutions and *CFL* numbers. In purely kinematic tests, smaller errors are obtained using CFL=1, since fewer substeps of the algorithm are necessary to obtain a given displacement. However, since the intended use of EBIT methods is advecting the interface in multiphase flows where the *CFL* has to be limited for stability reasons, the accumulation of errors will affect the performance.

**TABLE 1:** Surface error $E_{area}$, shape error $E_{shape}$, and order of convergence for two complete translations along the main diagonal, at different resolutions and *CFL* numbers.

$N_{x}$	CFL	$E_{area}$	$E_{shape}$	Order
64	1.0	2.89e−2	2.78e−2	2.17
	0.125	6.89e−1	7.01e−2	1.01
128	1.0	6.42e−3	1.23e−2	1.33
	0.125	3.43e−1	3.12e−2	1.00
256	1.0	2.56e−3	6.33e−3	0.93
	0.125	1.72e−1	1.55e−2	1.00
512	1.0	1.34e−3	3.57e−3	
	0.125	8.57e−2	7.87e−3	

### 3.2 Single vortex rotation

**FIGURE 4:**
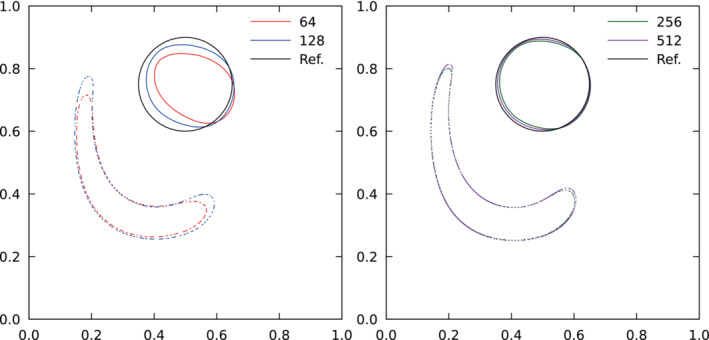
The interface at maximum deformation at t=1.0 (dotted line)and back to the initial position at t=2.0 (solid line) for the single vortex field test with T=2.0. [Colour figure can be viewed at wileyonlinelibrary.com]

The single vortex or “vortex‐in‐a‐box” problem has been designed to test the ability of interface tracking methods when the reference phase is highly stretched, see Reference 8. A circular shape of radius r=0.15 and center (0.5,0.75) is placed inside the unit box. The divergence‐free velocity u=(u,v) is obtained from the following stream function $\psi =\pi^{-1}\sin^{2}(\pi x)\sin^{2}(\pi y)\cos(\pi t/T),$ as $u_{x}=\partial \psi /\partial y$ and $u_{y}=-\partial \psi /\partial x$. On the sides of the box, homogeneous Dirichlet boundary conditions are imposed. The cosinusoidal time‐dependence slows down and reverses the flow, so that the maximum deformation occurs at t=T/2 and at time T the reference phase returns to its initial position with no distortion, allowing again to measure the error with (3) and (4), see Reference 9. For this test we use a constant time step Δt=0.0005. The position of the reference phase at t=1, corresponding to its maximum deformation, and at t=T=2 back to the initial position is shown in Figure 4. By refining the grid, the main fluid becomes thinner and more elongated at t=1, while tends to the reference initial shape at t=2. In Table 2 we report the surface error $E_{area}$, the shape error $E_{shape}$, and order of convergence at different grid resolutions.

**TABLE 2:** Surface error $E_{area}$, shape error $E_{shape}$, and order of convergence for the single vortex test with T=2.0, at different resolutions.

$N_{x}$	$E_{area}$	$E_{shape}$	Order
64	3.77e‐1	6.43e‐2	1.14
128	1.71e‐1	2.95e‐2	1.09
256	8.04e‐2	1.45e‐2	1.10
512	3.76e‐2	7.42e‐3

### 3.3 Zalesak's disk rotation

**FIGURE 5:**
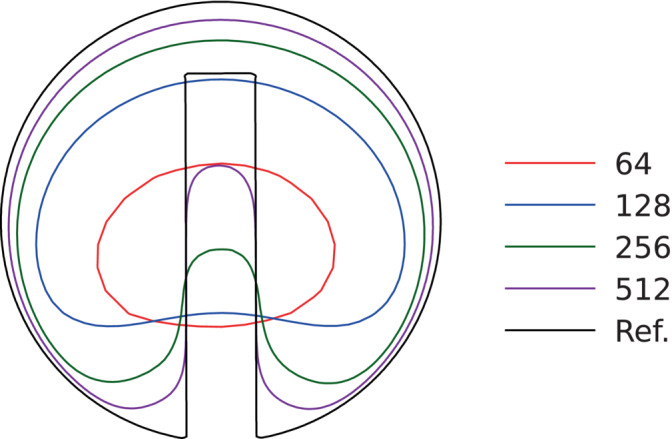
Initial (Ref.) and final shape for the Zalesak's disk after one rotation at T=1.0. [Colour figure can be viewed at wileyonlinelibrary.com]

In this test a notched circle of radius r=0.15 and center (0.5,0.75) is placed inside the unit box. The notched width is 0.05 and the length is 0.25. Imposing the constant velocity field (u,v)=(2π(0.5−y),2π(x−0.5)) the disk performs a full rotation around the box center and returns to the initial position at T=1.0. At the lowest resolution the notch disappears, while increasing the resolution the notch is maintained with smoothed corners (Figure 5). Interestingly, our method recovers final shapes that are symmetrical with respect to the notch vertical axis, which is not always observed in literature especially at low resolution, see References 10 and 11. In Table 3 we report the surface error $E_{area}$ and the order of convergence at different grid resolutions. The method exhibits a first‐order convergence rate upon grid refinement.

**TABLE 3:** Surface error $E_{area}$ and order of convergence for the Zalesak's disk rotation test with T=1.0, at different resolutions.

$N_{x}$	$E_{area}$	Order
64	7.56e‐1	0.87
128	4.12e‐1	1.27
256	1.71e‐1	1.36
512	6.69e‐2	

## 4 CONCLUSIONS

In this article, we have studied a new interface‐tracking method, where the interface is tracked by marker points located on the edges of the underlying grid. We have implemented the two‐dimensional version of the method using linear interface reconstruction. We have used three well‐known benchmark tests to validate the numerical method, recovering a first‐order convergence rate of the surface error, lower than the one obtained with other methods, such as VOF, level‐set, or isoAdvector.^4^, ^10^, ^11^ In future works we aim to use the EBIT method for multiphase simulations, developing models for topology changes and surface tension and extending the method to three dimensions.

## Data Availability

The data that support the findings of this study are available from the corresponding author upon reasonable request.
